# Pharmacovigilance in Vaccines: Importance, Main Aspects, Perspectives, and Challenges—A Narrative Review

**DOI:** 10.3390/ph17060807

**Published:** 2024-06-19

**Authors:** Katharine Valéria Saraiva Hodel, Bianca Sampaio Dotto Fiuza, Rodrigo Souza Conceição, Augusto Cezar Magalhães Aleluia, Thassila Nogueira Pitanga, Larissa Moraes dos Santos Fonseca, Camila Oliveira Valente, Cintia Silva Minafra-Rezende, Bruna Aparecida Souza Machado

**Affiliations:** 1 SENAI Institute of Innovation (ISI) in Health Advanced Systems (CIMATEC ISI SAS), SENAI CIMATEC University Center, Salvador 41650-010, Bahia State, Brazil; 2 Department of Medicine, College of Pharmacy, Federal University of Bahia, Salvador 40170-115, Bahia State, Brazil; 3 Department of Natural Sciences, Southwestern Bahia State University (UESB), Campus Vitória da Conquista, Vitória da Conquista 45031-300, Bahia State, Brazil; 4 Laboratory for Research in Genetics and Translational Hematology, Gonçalo Moniz Institute, FIOCRUZ-BA, Salvador 40296-710, Bahia State, Brazil; 5 Faculty of Veterinary and Animal Science, Federal University of Goiás, Goiânia 74690-900, Goiás State, Brazil; cintia.minafra@ufg.br

**Keywords:** pharmacovigilance, vaccines, adverse events, adverse events following immunization

## Abstract

Pharmacovigilance plays a central role in safeguarding public health by continuously monitoring the safety of vaccines, being critical in a climate of vaccine hesitancy, where public trust is paramount. Pharmacovigilance strategies employed to gather information on adverse events following immunization (AEFIs) include pre-registration data, media reports, clinical trials, and societal reporting. Early detection of AEFIs during clinical trials is crucial for thorough safety analysis and preventing serious reactions once vaccines are deployed. This review highlights the importance of societal reporting, encompassing contributions from community members, healthcare workers, and pharmaceutical companies. Technological advancements such as quick response (QR) codes can facilitate prompt AEFI reporting. While vaccines are demonstrably safe, the possibility of adverse events necessitates continuous post-marketing surveillance. However, underreporting remains a challenge, underscoring the critical role of public engagement in pharmacovigilance. This narrative review comprehensively examines and synthesizes key aspects of virus vaccine pharmacovigilance, with special considerations for specific population groups. We explore applicable legislation, the spectrum of AEFIs associated with major vaccines, and the unique challenges and perspectives surrounding pharmacovigilance in this domain.

## 1. Introduction

The monitoring of adverse events following exposure to pharmaceutical products is known as pharmacovigilance and is considered a fundamental tool for obtaining safe and effective drugs and vaccines [1]. The scope of pharmacovigilance activities is extensive, encompassing various strategies for collecting data on the safe use of medicines [2,3,4]. It is believed that vaccines are safer than drugs because they administer immunobiological agents to healthy individuals, while drugs are used for individuals with diseases; however, adverse events can occur in both scenarios [5]. The severity of these events is relative and can even lead to patient death [6]. Therefore, post-marketing monitoring of drugs and vaccines is of paramount importance for ensuring the safety of these products for the population [7]. After the vaccine is introduced into society, adverse reactions can occur, considering the genotypic diversity of the population and the existence of special groups such as elderly individuals, children, pregnant women, and immunosuppressed people [8]. These reactions can range from mild characteristics, such as transient pruritus, to severe anaphylactic reactions that may lead to death [9]. To ensure the safety of these products, a robust system for monitoring these occurrences is necessary.

In this context, notifications stand out as the primary means for consolidating the surveillance activity of drugs after their marketing [10]. In the case of vaccines, notifications made during the clinical trials phase are extremely important, enabling early analysis of the safety of the immunobiological product, thereby avoiding, or reducing the possibility of the emergence of serious and undesirable reactions in the population [11]. However, despite these efforts, ensuring complete and accurate reporting of adverse events remains a challenge. The swift identification and reporting of adverse drug reactions undeniably constitute important conditions for successful case investigations [12]. In hospital settings, the implementation of technological tools such as quick response (QR) codes or access links enable patients and/or companions to register adverse events more quickly and easily [13,14]. However, this is not the reality in most health facilities, especially in countries with economic constraints which face limitations in the services offered to the population [15,16]. This increased flexibility also facilitates decision-making in pharmacovigilance by managers and health professionals [17]. Effective pharmacovigilance practices are even more critical when considering that the perception of vaccine safety can sometimes be different from reality [1,18,19].

Pharmacovigilance efforts surrounding COVID-19 vaccines have been paramount in addressing public health concerns amidst the pandemic. There has been a significant emphasis on pharmacovigilance preparedness to achieve herd immunity through mass vaccination [20]. An extensive post-implementation pharmacovigilance study, utilizing VigiBase, a World Health Organization (WHO) global database, analyzed adverse event reports from more than 130 countries, ensuring comprehensive safety assessment [21]. The impact of misinformation on trust in health institutions and vaccine programs during the pandemic underlines the importance of ongoing safety monitoring [22]. The significance of mechanisms such as the National Pharmacovigilance System (NPS) ensures the safety of the COVID-19 vaccine’s post-market introduction [23]. Additionally, it is important to evaluate vaccine safety and efficacy among diverse populations through the analysis of adverse events following immunization (AEFIs) [24]. Together, these insights underscore the critical role of pharmacovigilance in mitigating risks and fostering public trust in COVID-19 vaccination programs worldwide.

Although healthcare facilities and professionals are continually encouraged to report adverse events related to vaccines, society could also contribute to these records. Experts believe that underreporting of adverse events occurs in all countries because individuals, rather than an automated case detection system, perform reporting [25]. Estimating underreporting by competent bodies, with values incorporated into the evaluation and monitoring of immunobiologicals through frequent updates, is indispensable [26,27]. Thus, the aim of this study is to explore the significance of pharmacovigilance in relation to vaccines, especially virus; examine the key aspects involved in monitoring the safety and efficacy of vaccines; and discuss future perspectives and challenges in this critical area of public health.

## 2. Overview of Vaccines

Vaccines, defined as biological agents eliciting an immune response to specific antigens from infectious pathogens, play a crucial role in suppressing the spread of various diseases [28]. The definition of a vaccine involves an immune-biological substance designed to produce specific protection against a given disease [29]. Vaccines have significantly impacted human health, playing a pivotal role in disease prevention and public health [30,31]. The history of vaccines traces back to the development of the smallpox vaccine by Edward Jenner in 1796 [32,33]. Jenner’s observations of cowpox lesions laid the foundation for vaccination, demonstrating immunity against smallpox [34]. The terms “vaccine” and “vaccinology” emerged following Jenner’s smallpox vaccine discovery, with Jenner often referred to as the “Father of Vaccinology” [35,36]. Since then, continuous efforts have been made to enhance vaccine safety and efficacy [37].

Vaccine development typically spans a prolonged process, averaging 10 years [38]. Nevertheless, the looming COVID-19 crisis engendered urgent hope for an expedited timeline at that time. Within this context, it is important to talk about Emergency Use Authorization (EUA), a strategy adopted by regulatory agencies for the use of unapproved medical products to diagnose, treat, or prevent serious or life-threatening diseases or conditions caused by threatening agents [39]. This initiative is adopted in response to an emergency health situation, when there are no suitable, approved alternatives available to control the disease [39]. The EUA has been widely used during the COVID-19 pandemic, especially for the development and accelerated availability of new vaccines for the disease [40].

The evolution of vaccine platforms over the last decade, enhanced nucleic acid-based, vectored, and recombinant protein vaccines, is noteworthy and the trajectory of these vaccines’ hinges on the success of all clinical trial developmental phases [41]. The SARS-CoV-2 pandemic has spurred unprecedented rapid action and necessitated the adaptation of production processes and leveraging preexisting data from related vaccines [42]. The commencement of clinical trials for ten COVID-19 vaccine candidates by June 2020 underscores the exceptional circumstances of this pandemic [38]. A brief timeline outlines key milestones in SARS-CoV-2 vaccine development, from the publication of the genetic sequence in January 2020 to the US Food and Drug Administration’s (USFDA) approval of mRNA vaccines in December 2020 [22,43]. The criteria of speed, scale-up manufacturing, and global access in vaccine development underscore that the remarkable efforts of researchers to expedite the process while ensuring efficacy and safety evaluations at each stage remain pivotal challenges [41]. The mRNA technology behind some COVID-19 vaccines has been extensively studied and intended for other purposes as well. This technology has shown potential in addressing challenges such as immune-mediated diseases and cancer [44].

Vaccines undergo rigorous testing processes, including large-scale clinical trials involving diverse populations to assess their efficacy rates, which are typically set at 50% or higher [45]. These trials, designed to identify any potential safety concerns, are essential steps before regulatory approval [46,47]. Efficacy is measured by comparing the occurrence of the ‘outcome of interest’, usually disease, between vaccinated individuals and those who receive a placebo [48]. While clinical trials provide valuable insights, real-world effectiveness is also assessed post-approval, considering a vaccine’s performance across various demographics and medical conditions [49,50]. Continuous monitoring post-approval further ensures the ongoing safety and the efficacy of vaccines [51,52,53]. Table 1 shows studies that demonstrated efficacy and safety of some vaccines.

**Figure 1:**
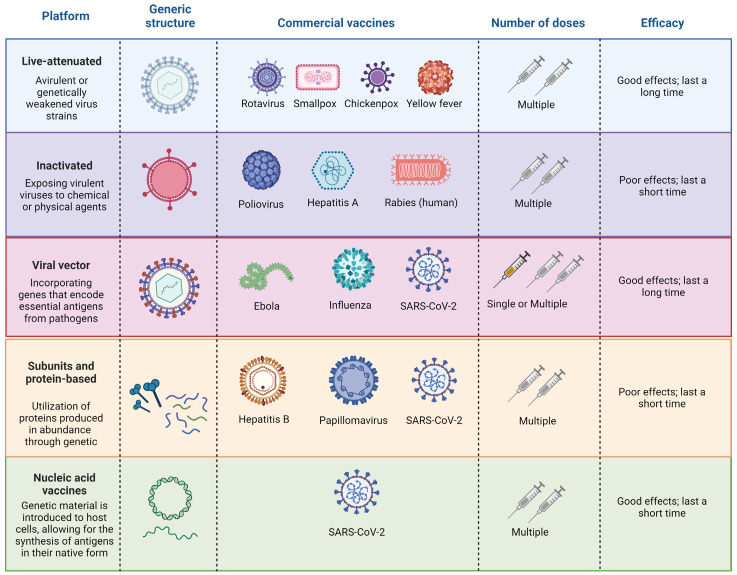
General information on the main viral vaccine platforms. Created with https://www.BioRender.com (accessed on 20 February 2024).

Administering the vaccine, known as vaccination, aims to protect individuals at risk, including children, elderly individuals, and those in disease-endemic areas [29]. Over the past 300 years, vaccines have made substantial contributions to human longevity and health, constituting a remarkable chapter in the history of science [72]. The field of vaccinology has evolved, encompassing immunology, molecular biology, and public health [73]. Different vaccine platforms contribute to the diversity of available vaccines (Figure 1) [29].

Despite substantial progress in vaccine research and development, challenges persist, especially in low- and middle-income countries (LMICs) [74,75]. Vaccination, recognized as one of the most cost-effective public health interventions, has yet to fully reach its target beneficiaries [76]. Approximately 20% of deaths among children under 5 years of age are attributable to diseases preventable by licensed vaccines [77]. The renaissance in vaccine research in the 1970s and 1980s led to the availability of various vaccines, emphasizing the critical roles of epidemiology and immunology in vaccine programs [74]. Briefly, live-attenuated vaccines comprise a modified form of the living virus, intentionally weakened to the extent that it no longer induces severe illness in individuals with robust immune systems [78,79]. Inactivated virus vaccines are typically produced by exposing virulent viruses to chemical or physical agents, such as formalin or β-propiolactone [80]. This exposure is designed to destroy the infectivity of the virus while retaining its immunogenic properties. Viral vectors are intricately engineered to facilitate the introduction of target genes that encode crucial antigens of pathogens [81]. Through genetic manipulation, these vectors are designed to serve as carriers, delivering specific genetic material into host cells [82]. Subunit vaccines rely on overexpressed proteins derived from genetically modified *Escherichia coli* (*E. coli*), serving as a link between natural and recombinant DNA technology [83]. Nucleic acid vaccines, a newer technology between live and killed approaches, use DNA or messenger RNA to produce antigens, presenting them in their native form and eliciting diverse immune responses without the risk of infection [83,84].

Due to their huge reduction in infectious disease-related morbidity and death, vaccines have had a major effect on public health [30]. However, there is growing recognition of the broader economic and social benefits of vaccines [31,85]. These include the prevention of long-term disability, reduced healthcare costs associated with treating vaccine-preventable diseases, and the preservation of societal productivity [31]. Despite the undeniable benefits, it is important to acknowledge that vaccines, like any medical intervention, can have side effects. While these side effects are typically mild and transient, they are essential indicators that the body’s immune system is responding appropriately to the vaccine [31]. It is crucial to communicate both the benefits and potential risks of vaccination to the public to ensure informed decision-making and maintain trust in vaccination programs [86].

## 3. Pharmacovigilance

Pharmacovigilance is as a crucial process in guaranteeing the safety and efficacy of vaccines [87]. This multidisciplinary research area involves the ongoing monitoring, evaluation, and prevention of adverse drug events, encompassing vaccines [88]. Throughout history, vaccines have played a pivotal role in disease prevention and public health promotion. However, it is essential to acknowledge that, like all medications, vaccines may induce adverse effects in some individuals [89]. Pharmacovigilance becomes paramount in the timely and effective detection, assessment, and prevention of these adverse effects [90].

A fundamental aspect of vaccine pharmacovigilance is the continuous monitoring of safety data [91]. This approach entails gathering information from diverse sources such as medical records, patient reports, and controlled clinical trials [92]. Regular analysis of these data is conducted to identify safety patterns and evaluate the benefit–risk balance of a specific vaccine [22]. With the growing number of WHO-recommended vaccines, there is a pressing demand for more cost-effective products that are easier to deliver and that enhance the safety and acceptability of vaccines at the point of delivery [93].

### 3.1. Overview of Pharmacovigilance

Active and passive surveillance are two primary methods used in monitoring public health. Active surveillance involves dedicated staff members who regularly reach out to healthcare providers or the population to gather information about health conditions [94]. This approach ensures the collection of the most accurate and timely data, making it highly reliable, though it comes with significant costs. In contrast, passive surveillance relies on reports voluntarily submitted by hospitals, clinics, public health units, and other sources [95]. This method is cost-effective and capable of covering large areas, providing essential insights into community health. However, the quality and timeliness of data in passive surveillance can be inconsistent, as it depends on the voluntary participation of various institutions and individuals [96]. Despite these differences, both methods are crucial for comprehensive public health monitoring, with active surveillance offering precision and immediacy, while passive surveillance provides broad, economical coverage.

Pharmacovigilance involves monitoring, detecting, evaluating, and preventing adverse drug-related effects [97]. To efficiently carry out this task, several tools aid in collecting, analyzing, and reporting information on adverse drug events [98]. These tools include the following:

(i) Pharmacovigilance information systems: these systems allow the collection, storage, analysis, and communication of information on adverse drug events;
(ii) Pharmacovigilance databases: these databases serve as reliable sources of information on adverse drug events;
(iii) Adverse event tracking applications: these tools assist in monitoring and tracking adverse drug events;
(iv) Statistical analysis: this technique is used to evaluate data collected on adverse drug-related events.

After analyzing the data, pharmacovigilance is responsible for disseminating all the obtained information to the community, particularly the incidence and severity level of each toxic event [99]. The primary tool of pharmacovigilance for recording and storing adverse drug reaction reports in a country is crucial [100]. The quality and robustness of this system enable effective actions, including the accurate recording of reports, easy access to data by health managers, and the implementation of practices to prevent serious reactions from the use of medicines [101].

Causality assessment is crucial in medical practice as it helps identify drugs responsible for adverse drug reactions (ADRs), potentially saving lives and preventing further harm. The assessment follows four key principles: the temporal relationship between the drug and the reaction, biological plausibility, de-challenge, and rechallenge [102]. Pharmaceutical sponsors are frequently required by regulators to employ structured techniques for assessing causation, which fall into three categories: probabilistic methods (e.g., Bayesian), expert judgment (e.g., WHO-UMC system), and algorithm-based approaches (e.g., Naranjo scale) [103]. These methods help address the challenge that most ADR reports involve suspected, not confirmed, reactions. The WHO-UMC system, developed with input from national centers, combines clinical and pharmacological data to assess causality, considering both case history and documentation quality [104]. MedDRA, a standardized medical terminology dictionary, supports this process through all stages of drug development and post-marketing surveillance, facilitating the registration, documentation, and monitoring of medicinal products [105].

### 3.2. Pharmacovigilance of Vaccines

#### 3.2.1. Legislation and Responsible Authorities

Pharmacovigilance is a globally regulated discipline with significant oversight from three major institutions: the European Medicines Agency (EMA), the Food and Drug Administration (FDA), and the Pharmaceuticals and Medical Devices Agency (PMDA) [106]. Additionally, three internationally recognized pharmacovigilance systems, namely the European Union (EU) pharmacovigilance system, the WHO Uppsala Monitoring Center system, and the International Conference on Harmonization (ICH) system, play pivotal roles in shaping global pharmacovigilance standards [106]. The influence of developed countries in steering worldwide pharmacovigilance can be attributed, in part, to their commendable responsiveness, monitoring capabilities, and support for health-related queries concerning medication safety [107,108]. Table 2 summarizes the main systems used in Brazil, the United States (US), Japan, Australia, as well as European and African countries to report vaccine adverse reactions and the regulatory agencies responsible for monitoring those notifications.

Certain countries have specific protocols for post-vaccination reaction notifications. In Australia, healthcare professionals are mandated to report medicine or vaccine events either online or by utilizing a downloadable template [106]. Chile, which joined the Pharmacovigilance Program initiated by the WHO and the Uppsala Monitoring Center (UMC) in 1972, implemented pharmacovigilance for SARS-CoV-2 in December 2020 [121]. This effort involves monitoring patients and potential reactions following COVID-19 vaccination. The United Kingdom established a Committee on the Safety of Medicines (CSM) after the thalidomide disaster, focusing on monitoring unexpected reactions and requiring entities to report adverse reactions, especially for new medicines and vaccines [122]. To enable this reporting system, the UK’s Medicines and Healthcare products Regulatory Agency has adopted the Yellow Card system for reporting suspected adverse events to different pharmaceutical or biomedical products, including vaccines [123]. In the United States, pharmacovigilance has been practiced since 1938 under The US Federal Food, Drugs, and Cosmetics Act [124]. The thalidomide tragedy of 1960 reinforced the need for improved monitoring of adverse reactions in new products. Presently, the Vaccine Adverse Event Reporting System (VAERS) allows the public to report adverse reactions to vaccines, with WONDER, an online database, facilitating inquiries about vaccine adverse events [106,125].

In 2021, the Brazilian Ministry of Health instituted the National Interinstitutional Committee for Pharmacovigilance of Vaccines and other Immunobiologicals, known as CIFAVI [126]. The committee’s primary objective is to assess the technical and scientific aspects of adverse events stemming from the use of vaccines, while also considering the responsibilities of public agencies involved in pharmacovigilance initiatives [127]. The CIFAVI is tasked with investigating the causes behind adverse postvaccination events, overseeing the progression of studies on these reactions, and recommending additional investigations, particularly for severe and rare cases [126,127].

In the EU, the standardization of pharmacovigilance was effectively implemented in July 2012 [128]. This consolidation of requirements for registration and authorization holders ensures efficient sanitary control mechanisms for reporting and monitoring adverse reactions [129]. The European Medicines Agency provides comprehensive information on good practices in pharmacovigilance (GVP) on its website [130]. GVP is organized into chapters containing definitions, templates, guides, and actions aimed at promoting the proper practice of pharmacovigilance, including a section on vaccines for prophylaxis against infectious diseases. The term adverse event following immunization (AEFI) is defined by the EMA as “any untoward medical occurrence following immunization without necessarily having a causal relationship with the vaccine’s usage” [5]. AEFI is synonymous with the term post-vaccination adverse events (PVAE) used in Brazil. Notably, the WHO classifies AEFIs into five categories: vaccine product-related reactions, vaccine quality defect-related reactions, immunization error-related reactions, immunization anxiety-related reactions, and coincidental events [131].

The EU’s involvement in vaccine pharmacovigilance encompasses various stakeholders, including individuals receiving the vaccine, parents/guardians of those vaccinated, healthcare professionals directly or indirectly involved in vaccination, registration holders, clinical trial sponsors, health regulatory authorities, and public health authorities recommending vaccination programs [132]. Key entities overseeing the reporting of post-immunization events include the European Medicines Agency, the European Centre for Disease Prevention and Control (ECDC), and the WHO.

In 2009, Japan’s PMDA launched the MIHARI project to establish an innovative framework for pharmacoepidemiological drug safety assessments [133] for: (i) ensuring accessibility to existing databases, laying the groundwork for subsequent analyses; (ii) systematically characterizing each database through simple tabulation and validation studies, providing a robust foundation for further investigations; and (iii) employing pharmacoepidemiological methods to conduct pilot studies, specifically targeting risk evaluations associated with anticipated adverse drug events, aligning with information found in package inserts or reported in prior studies [115].

The Pharmacovigilance Africa (PAVIA) initiative, funded by the European Developing nations Clinical Trials Partnership (EDCTP), aims to enhance pharmacovigilance (PV) in Tanzania, Ethiopia, Nigeria, and Eswatini [117]. PAVIA will leverage collaboration among institutions in Europe and Africa, focusing on improving adverse event reporting procedures, especially in relation to new medications for multidrug-resistant tuberculosis (MDR-TB) [134]. Additionally, the initiative will provide training and expertise to promote integration between disease control programs and regulatory authorities [135].

In Australia, the administration of the Act’s provisions falls under the purview of the Therapeutic Goods Administration (TGA), a division of the Commonwealth Department of Health and Ageing [119]. Responsible for overseeing the production, distribution, import, export, and manufacturing of medicinal products, the TGA plays a critical role in ensuring the safety and efficacy of healthcare goods available to Australians [120]. The TGA operates with efficiency and timeliness to regulate therapeutic products, thereby safeguarding and enhancing community health across the nation [136]. Central to its regulatory efforts is the maintenance of the Australian Register of Therapeutic Goods (ARTG), a comprehensive database containing information about every medicinal product imported into, supplied in, or exported from Australia.

In addition, it is important to note that there are differences when it comes to pharmacovigilance legislation between lower middle-income countries (LMIC) and high-income countries (HIC). Part of the approach adopted by health authorities in LMICs is to carry out educational activities for health workers on the need to report adverse events [137]. Although this strategy has its due importance, it is noted that there is a need to promote improved passive and active surveillance, which can be related to the establishment of electronic and online reporting systems, as well as national incentive programs [137]. A study conducted in Sierra Leone showed that although the national regulatory authority has structures and processes capable of supporting pharmacovigilance initiatives, health facilities and health programs have an urgent need to fully operationalize their structures [138]. On the other hand, HICs generally have robust pharmacovigilance systems, most of which follow the guidelines established by the EMA [106].

#### 3.2.2. Monitoring Special Populations

The pharmacovigilance of special groups, composed of children, pregnant women, elderly adults, and individuals with chronic diseases or immunosuppression, is a critical aspect of ensuring vaccine safety and efficacy, particularly in populations with unique considerations and vulnerabilities [53,139]. This occurs for different reasons, including the particularities related to the pharmacokinetics and pharmacodynamics of immunization in these individuals [140]. Miller [141] emphasized the importance of understanding the degree of immune compromise and recovery in these populations, acknowledging that higher vaccine doses or more frequent boosters may be needed.

Vaccination in pregnant and breastfeeding mothers is a widely used strategy to reduce the risk of several infectious illnesses infecting mothers and their unborn children [142,143]. This underscores the importance of considering the unique aspects of pregnancy-related immunology in vaccine pharmacovigilance efforts. The physiological changes associated with pregnancy, leading to an elevated risk of severe disease, are further accentuated by an altered state of immune responsiveness due to adaptive immune responses by the mother to tolerate the fetus and vice versa [144]. While immunogenicity studies suggest comparable responses in pregnant and nonpregnant women, caution is advised, with vaccination ideally deferred until the second or third trimester to minimize theoretical fetal harm [141].

Older adults, a rapidly growing population, face increased susceptibility to infections due to immunosenescence [143,145]. Immunosenescence affects both innate and adaptive immunity, leading to a decline in responses to both pathogens and vaccines [146]. This decline in immune function necessitates special attention in vaccine development and administration to protect against age-related diseases [147]. Consequently, older adults are typically more susceptible to vaccine-preventable diseases, and the severity of disease can be more pronounced in older adults than in younger individuals [148,149]. This finding shows the importance of tailored vaccine strategies for the elderly population.

Approximately one in three adults worldwide has at least one chronic illness, and in the US, this figure is as high as 45% of the entire population [150]. These individuals necessitate tailored immunization strategies due to their heightened susceptibility to infections and potential contraindications for live vaccines, especially immunosuppressed individuals [151]. Immunocompromised patients represent a diverse group with varying degrees of immunosuppression [152]. Individuals with primary disorders of the immune system face the challenges of inherited conditions that may affect various components, such as humoral, cellular, and phagocytic functions [141,153]. These disorders share a common feature—susceptibility to infections caused by various organisms, some of which can be prevented through immunization, contingent on the specific type of immunodeficiency [141,154]. Understanding the specific immune deficiency associated with underlying conditions is crucial for tailoring vaccination strategies to mitigate infection risks. Guidelines provided by the Infectious Disease Society of America, play a crucial role in shaping vaccination strategies for immunocompromised individuals [155]. Specifically, live-attenuated vaccines, are contraindicated in actively immunosuppressed patients [156]. This precaution is rooted in the increased risk of developing the targeted disease and the potential for prolonged shedding of the vaccine virus [143].

The realm of immunizations in special populations underscores the critical importance of a nuanced understanding of underlying diseases and their potential impact on the immune system’s ability to mount an effective antibody response to vaccines. The susceptibility of these groups to more frequent and more serious adverse events makes the focus of pharmacovigilance crucial in terms of contributing to the development of safer pharmaceutical strategies.

## 4. Vaccines and Adverse Reactions after Immunization

Monitoring adverse reactions to vaccines is crucial for ensuring their safety and efficacy [157]. The primary goal of such monitoring is to identify and assess adverse events linked to drugs, medical devices, vaccines, and other health products regulated by health authorities [90]. As an integral part of the evaluation process for vaccine safety and efficacy, monitoring adverse reactions plays a pivotal role in leveraging this information to protect public health [27,158,159].

Monitoring serves to detect any issues with vaccines, prompting potential changes in administration protocols, vaccination recommendations, or even the withdrawal of a vaccine from the market [160].

The following are some approaches for monitoring adverse reactions to vaccines:

(i) Voluntary reporting systems [161,162]: a prevalent method in which healthcare professionals, patients, and vaccine manufacturers are encouraged to report adverse events to regulatory bodies;
(ii) Clinical Trials [163]: participants are closely monitored for vaccination-related adverse reactions;
(iii) Vaccination registries [164]: systems that collect information on vaccine administration and associated adverse events;
(iv) Public health research [165]: cohort studies and randomized controlled trials may offer insights into long-term adverse reactions related to vaccination.

Voluntary reporting systems for adverse drug reactions (ADRs) have been established in many Western nations since the early 1960s, enabling doctors and pharmacists to report suspected ADRs [161]. Despite efforts to simplify the reporting process, underreporting remains a significant concern, with only a minimal number of ADRs genuinely reported to pharmaceutical firms and national reporting centers [166]. Reasons for underreporting include bureaucratic hurdles, time constraints, and ignorance about reporting procedures [167].

Despite the existence of reporting systems, a significant underreporting of AEs continues to be a major challenge in pharmacovigilance. Studies estimate that over 90% of AEs go unreported by healthcare professionals (HCPs) [168,169]. This underreporting can have serious consequences, delaying the detection of safety concerns and hindering the proper evaluation of drug risks. The reasons for underreporting are multifaceted and can be categorized into psychological obstacles faced by HCPs and systemic problems within healthcare systems [169,170].

Psychological obstacles can include fear of professional consequences, such as accusations of incompetence or malpractice. Additionally, HCPs might experience feelings of indifference, diffidence, or insecurity when reporting AEs [166]. A lack of knowledge or understanding of pharmacovigilance can also lead to complacency, where HCPs fail to recognize or report AEs altogether [171]. Systemic problems that contribute to underreporting include a lack of training for HCPs on how to identify and report AEs [169]. Inertia and limited time constraints can also discourage reporting. Furthermore, cumbersome reporting processes and a lack of clear feedback on submitted reports can further demotivate HCPs.

Underreporting is also observed in the case of spontaneous notifications, one of the pharmacovigilance methodologies [172]. This limitation, coupled with the fact that the reports submitted are often of poor quality, means that spontaneous notifications present challenges. In many cases, there is a lack of data that would allow for better clarification of the adverse event [4]. Despite these challenges, spontaneous reporting by healthcare professionals remains a cornerstone of pharmacovigilance systems, offering valuable real-world data to address medication safety issues and mitigate the impact of ADRs on society [4].

Pharmacovigilance in clinical trials plays a pivotal role in ensuring the safety of participants involved in the research [173]. It encompasses the ongoing monitoring of the benefit:risk relationship of investigational pharmaceuticals administered during the trial [46,174]. Sponsors and investigators have a responsibility to promptly report any adverse events or changes in this relationship to relevant authorities and ethics committees [175,176]. Compliance with regulatory standards, such as ICH GCP and USFDA guidelines, is essential to provide a robust evidence base for medication approvals while prioritizing patient safety [177]. Additionally, proactive safety reviews during clinical trials help mitigate risks and contribute to the overall success of drug development efforts [178].

Pharmacovigilance and vaccination registries serve as essential resources for evaluating vaccine uptake and impact within populations [164,179]. These population-based registers enable the assessment of dynamic vaccination programs, monitoring of time trends, and identification of sub-populations with low vaccination coverage [180]. Continuous monitoring of the quality of these registers is crucial for ensuring reliable evaluation of vaccine uptake and impact at a national level [27,181]. Additionally, the documentation generated through this process can inform improvements in the design and quality of other vaccination or healthcare registers, contributing to more effective public health interventions [180].

Adverse reactions to vaccines can vary depending on the type of vaccine and the individual receiving it [182]. Common reactions include pain, swelling, and redness at the injection site, as well as fatigue, headache, muscle aches, chills, fever, nausea, and swelling of the lymph nodes [183]. These reactions are typically mild and tend to resolve within a few days [184]. It is important to mention that from this perspective, adverse events following immunization (AEFIs) are widely used and can be defined as any medical occurrence following immunization that does not necessarily have a causal relationship with the administration of the vaccine [185].

In this context, it is important to note that the literature has reported a difference between the frequency of adverse events following vaccination when comparing men and women [186]. As vaccination rates are similar between both sexes, the differences observed between them are related to biological issues. When it comes to viral vaccines, the intensity and frequency of adverse events following vaccination is usually higher among females [187]. The same trend has been reported for COVID-19 immunization campaigns [188]. These findings point to the need to monitor targeted adverse events, considering the possible differences between the sexes.

Adjuvants and other vaccine components, such as preservatives and antibiotics, rather than the active ingredient itself usually trigger allergic reactions to vaccines [189]. These reactions are considered idiosyncratic and may manifest as hives, rashes, swelling, or itching in response to various vaccines, including the measles, mumps, and rubella (MMR) vaccine; the flu vaccine; and the chickenpox vaccine [190,191]. Recently, for the COVID-19 vaccine, in addition to the common effects, some individuals have reported more serious side effects, including allergic reactions, myocarditis (inflammation of the heart muscle), and thrombosis (blood clot formation) [192,193]. It is important to note that these cases are extremely rare. Given the relevance to public health and the number of published articles involving the analysis of adverse events following vaccination, the following topics will present a more in-depth analysis of the profiles of COVID-19, polio, influenza, and hepatitis B vaccines.

### 4.1. COVID-19 Vaccines

Vaccines designed to prevent SARS-CoV-2 infection are widely acknowledged as the most promising strategy for mitigating the COVID-19 pandemic caused by the 2019 coronavirus disease [194]. Globally, by the end of December 2023, more than 13 billion doses of COVID-19 vaccines had been administered [195]. The rapid spread of SARS-CoV-2, an unprecedented virus, and the need to develop new vaccines quickly to support the epidemiological control of the disease have meant that the pharmacovigilance infrastructure has had to adapt and include tools to enable risk and benefit analyses [196].

The primary antigenic target for COVID-19 vaccines is the surface spike protein, which binds to the angiotensin-converting enzyme 2 (ACE2) receptor on host cells, initiating membrane fusion [197,198]. Antibodies that bind to the receptor-binding domain of the SARS-CoV-2 spike protein play a crucial role in preventing its attachment to host cells and neutralizing the virus [42,199]. Various platforms have been employed in the development of COVID-19 vaccines [200,201]. Some use traditional approaches, such as inactivated viruses or live-attenuated viruses, which have proven successful in influenza [202] and measles vaccines [203], respectively. Other approaches utilize newer platforms, including recombinant proteins [204] (as seen in human papillomavirus vaccines) and vectors [205] (utilized in Ebola vaccines). Additionally, platforms such as RNA and DNA vaccines, which were previously unused in licensed vaccines, are being explored [206,207].

Currently, the recommended vaccination strategy for individuals aged six years and older involves receiving at least one dose of the bivalent mRNA vaccine against SARS-CoV-2 [208]. The single dose regimen of the mRNA vaccine is generally considered sufficient to enhance preexisting immunity in vaccinated individuals [209]. However, immunocompromised individuals and those aged 65 and older may opt for a second dose [210]. Children aged between six months and five years should adhere to a vaccination schedule tailored to their age and the type of vaccine [211]. Notably, monovalent mRNA vaccines are no longer recommended [212].

#### 4.1.1. Adverse Reactions

Vaccines designed to prevent SARS-CoV-2 infection represent a promising strategy in curtailing the COVID-19 pandemic [213]. Currently, several vaccine options are available globally [214]. There are noteworthy adverse effects of COVID-19 vaccines, including allergic reactions and vaccine-induced immune thrombotic thrombocytopenia, discussed within the scientific community [215]. Other adverse effects, though infrequent, include ocular adverse events [216], myocarditis [217], pericarditis [218], and Guillain–Barré syndrome [219]. Severe allergic reactions (anaphylaxis) have been reported in response to COVID-19 vaccines [9], including those developed by Pfizer-BioNTech, Moderna, and Johnson & Johnson [220]. It is crucial to emphasize that anaphylaxis following vaccination is extremely rare, and the overall benefits of vaccination outweigh the associated risks [221,222]. Table 3 describes the available COVID-19 vaccines and their rare adverse effects.

#### 4.1.2. Special Populations

Numerous studies have identified risk factors contributing to clinical deterioration in patients infected with SARS-CoV-2 [240]. The key risk factors associated with the development of severe coronavirus disease are outlined in Table 4 and include advanced age (>65), obesity, type 2 diabetes mellitus, hypertension, and other cardiovascular diseases [241,242,243,244]. These studies consistently highlight that the presence of these conditions correlates with a more adverse clinical outcome of the disease. Consequently, the prioritization of the COVID-19 vaccine has become justified, as it serves to prevent SARS-CoV-2 infection in individuals with these identified risk factors [245]. In Nepal, a study of vulnerable populations after the first dose of Covishield showed no significant association between the occurrence of events and comorbid diseases [246]. Another analysis conducted in Saudi Arabia indicated that the presence of underlying comorbidities such as hypertension, diabetes, pulmonary diseases, and cardiovascular diseases did not emerge as a significant risk factor for the development of systemic adverse events following immunization with either the first or second dose of the Comirnaty (mRNA) and Covishield (recombinant) vaccines [247]. These results suggest the safety of approved COVID-19 vaccines, even among at-risk groups.

### 4.2. Polio Vaccine

Poliomyelitis, commonly known as polio, is a vaccine-preventable disease caused by highly infectious poliovirus and is primarily transmitted through the fecal–oral route [59]. By targeting mainly children under the age of 5 [258], the virus can lead to an acute nonspecific illness, with 72% of infected children remaining asymptomatic but capable of shedding the virus [259]. The most severe consequences arise from invasion of the central nervous system by the virus, resulting in significant morbidity, including paralysis and, in 1 in 200 cases, respiratory failure [59,260]. With no cure for polio, prevention is of utmost importance [261].

Two main types of polio vaccines have played a pivotal role in disease eradication efforts [262]. Jonas Salk pioneered the inactivated or killed vaccine in 1955 [263], followed by Albert Sabin’s live, attenuated or weakened vaccine in 1961 [264]. These vaccines contributed to the successful eradication of polio in the United States by 1979 [265]. Currently, the CDC recommends the trivalent inactivated polio vaccine as part of routine childhood vaccinations [59].

Three serotypes of poliovirus exist, with wild-type 2 (WPV2) and wild-type 3 (WPV3) considered eradicated, but wild-type 1 (WPV1) still circulating and causing disease in Afghanistan and Pakistan [266,267]. Two vaccines are commonly used to protect against polio: the live-attenuated oral poliovirus vaccine (OPV) and the inactivated poliovirus vaccine (IPV) [266,268]. In most countries, a combination of bivalent OPV (type 1 and type 3) and IPV is employed [269]. OPV, being more cost-effective, replicates in the recipient’s gut, eliciting superior primary intestinal immunity compared to IPV [270]. When administered orally as drops, OPV eliminates the need for trained health workers during vaccination, making it a practical and cost-effective solution, particularly in resource-constrained regions [271,272]. The convenience of oral administration facilitates broader coverage and community-wide immunity [273]. This method also enhances the efficiency of vaccination campaigns, enabling rapid responses to outbreaks and reaching populations in remote areas [274].

The IPV, known as poliovirus vaccine inactivation, is the primary formulation used in the United States. Developed using formaldehyde-inactivated virus grown on monkey kidney tissue culture, the vaccine is a single-disease immunization [275,276]. It contains a preservative and trace amounts of polymyxin B, streptomycin, and neomycin [277]. Additionally, it is available in combination with other vaccines in formulations such as DTaP/IPV/Hib, DTaP/Hep B/IPV, and DTaP/IPV [278]. Different countries, such as Brazil, the United States, and Canada, recommend routine immunization of babies and children against polio [279].

#### 4.2.1. Adverse Reactions

Adverse reactions following routine immunization, including polio vaccination, are generally rare but can vary in severity [280,281]. Serious reactions (Table 5) such as hypersensitivity, anaphylaxis, paralysis, and seizures are infrequent, occurring at a rate of 1 per million doses administered [282].

Frequently, individuals may encounter localized reactions to the vaccine, presenting as redness or discomfort at the injection site [287]. Infrequent minor adverse reactions include irritability, fatigue, loss of appetite, fever, and vomiting [59]. The most common cases of AEFI for both types of IPV are generally classified as minor, vaccine product-related reactions [288,289]. These reactions are typically mild, self-limited, and relatively minor [286]. Fever is the most frequent symptom and/or sign observed in AEFI patients associated with both types of IPV, consistent with results from surveillance reports and clinical trials [289].

In areas characterized by low immunization rates and the administration of the oral polio vaccine, there is a slight risk of vaccine-derived poliovirus (VDPV) [290]. This risk arises from the live-attenuated virus potentially acquiring virulence, presenting an infectious threat [291]. Notably, VDPV is not associated with IPV, the exclusive polio vaccine used in the United States for routine childhood vaccinations since 2000 [59].

#### 4.2.2. Special Populations

The administration of the polio vaccine, particularly in special populations, follows specific recommendations provided by authoritative health organizations [59]. On a global scale, the World Health Organization (WHO) underscores the importance of immunizing all children worldwide against polio, urging countries to achieve and maintain high vaccination coverage [292]. Specific intervals between doses are recommended, particularly for infants traveling to endemic countries or at risk of exposure to wild poliovirus [293]. For routine childhood immunization against polio, the recommended regimen consists of a 4-dose series of IPV, administered at 2 months, 4 months, 6 to 18 months, and 4 to 6 years of age [294]. The third and fourth doses are to be separated by at least six months, and some children, due to the use of combination vaccines, may safely receive up to five doses [280].

Children under the age of five are more susceptible to polio, making them a group with more effort put into vaccination campaigns. A study carried out in India showed that fever and swelling were the most commonly reported AEFIs for children up to 2 years of age [295]. In Pakistan, one of the reasons for refusing routine childhood vaccination is associated with fear of AEFI and the severity of adverse events [296]. However, evidence shows that polio vaccines (IPV and OPV) are among the most vulnerable groups have a safe profile [297].

### 4.3. Influenza Vaccines

Influenza, caused by viruses belonging to the *Orthomyxoviridae* RNA family, is a globally significant respiratory disease that includes influenza A, influenza B, influenza C, and influenza D [298,299]. These viruses, which are transmitted through various means such as vomiting, coughing, and sneezing, express critical antigens, hemagglutinin (HA) and neuraminidase (NA), which are pivotal for their virulence [300]. Influenza viruses infect the respiratory tract, causing symptoms ranging from mild upper respiratory issues to severe pneumonia [300,301,302]. The influenza vaccine induces immunity by eliciting antibodies targeting these antigens [303].

Influenza is an enveloped RNA virus that causes a spectrum of respiratory symptoms, and vaccination remains a key preventive measure [302,304]. The history of the influenza vaccine dates back to World War II, with early attempts leading to the first licensed vaccine in 1945 [305]. Modern influenza vaccines, mostly administered intramuscularly, aim to provide effective protection against evolving virus strains, preventing severe complications and reducing the impact of annual epidemics [306,307]. Annual vaccination is recommended due to waning immunity and the need for updated antigens [302,308].

The live-attenuated influenza vaccine (LAIV) is administered intranasally, inducing both mucosal and systemic immunity against native hemagglutinin and neuraminidase glycoproteins [309]. This unique administration stimulates specific nasal and serum antibodies and T-cell responses, closely resembling those generated by natural influenza infection [310]. Notably, LAIV can elicit a more robust immune response, offering protection not only against the included vaccine strains but also against mismatched strains [309,311]. Additionally, LAIVs include cold-adapted, temperature-sensitive, attenuated influenza viruses, designed to replicate moderately, preventing them from reaching concentrations that induce disease, further enhancing their safety profile (Fischer 2020).

Trivalent inactivated influenza vaccines protect against two influenza A strains (H1N1 and H3N2) and one influenza B strain, while quadrivalent vaccines cover an additional influenza B strain [312]. Quadrivalent vaccines (QIVs), containing representatives of influenza A and B strains, are standard, with antigens regularly updated based on WHO recommendations [313,314]. The predominant approach for influenza vaccination worldwide involves the use of inactivated virus vaccines, owing to their elevated safety profile and relatively economical production costs [307,315]. This strategy accounts for the largest share of the global flu vaccine market [307,316]. Notably, they are three main types of inactivated influenza virus (IIV): whole-virus inactivated vaccines, split-virus inactivated vaccines, and subunit inactivated vaccines, each of which are designed to confer robust immune responses [307]. Typically, the virus is cultivated in embryonated chicken eggs or cultured mammalian cells to produce this type of vaccine [317,318]. IIV has demonstrated efficacy in eliciting both local and systemic immunity [319]. However, periodic booster vaccinations may be required to sustain optimal antibody titers [320].

#### 4.3.1. Adverse Reactions

Influenza vaccination, while generally considered safe and effective, may be associated with various adverse events [321]. These events can manifest as both injection site reactions and systemic symptoms [287]. Commonly reported reactions include fever, irritability, drowsiness, myalgia, and upper respiratory symptoms when administered via nasal spray [300]. Notably, severe allergic reactions such as urticaria or anaphylaxis have been documented [322,323].

Adverse event reports are categorized as serious or not serious, with serious events involving outcomes such as death, life-threatening illness, hospitalization, permanent disability, or those requiring medical intervention [324]. It is important to emphasize that the submission of an adverse event report does not imply a causal relationship to vaccination [5]. Temporally associated adverse events of interest include myalgia, cough, rash, and headache, with more severe reactions such as febrile convulsions and Guillain–Barré syndrome also recorded in some cases [325] (Table 6).

#### 4.3.2. Special Populations

Influenza vaccination is a crucial preventive measure recommended by the CDC for individuals above six months of age without contraindications [300,336]. The efficacy of the influenza vaccine is particularly notable in children over two years of age and healthy adults, making it a key strategy for preventing and controlling influenza [300]. Complications arising from influenza, especially in young children, older adults, pregnant individuals, and those with specific underlying conditions, emphasize the importance of targeted vaccination efforts [302,337,338]. The WHO recommends seasonal influenza vaccination, which is the highest priority for pregnant women [339]. Additionally, individuals, in no particular order of priority, include children aged 6 months to 5 years, elderly individuals (>65 years old), individuals with specific chronic medical conditions, and healthcare workers [305,338].

The results reported by Carreras et al. [340], who monitored possible AEFI a with tetravalent vaccine in pregnant women in Spain showed that there were no reports of spontaneous abortions, prematurity, or fetal malformations, and that the rate of AE reporting was lower among pregnant women than among nonpregnant women. The study by Daley et al. [341] evaluated data between 2003 and 2013 on LAIV immunization considering individuals between 2 and 17 years of age in the United States. The study showed that the most reported serious adverse events were anaphylaxis and syncope, although they were extremely rare, which reinforces the vaccine’s safety profile. A study carried out in Brazil analyzed AEFI after doses of the monovalent version of the vaccine were administered to healthcare workers, and the most frequent AEs were fever, headache, myalgia, and pain at the injection site, with no reports of serious occurrences [342].

### 4.4. Hepatitis B Vaccine

Hepatitis B vaccination plays a pivotal role in preventing active infection with the hepatitis B virus (HBV), a highly infectious pathogen that poses serious risks, including chronic liver failure and hepatocellular carcinoma [343]. The vaccine, introduced in 1981 and later refined with a recombinant version in 1986, marked a significant shift in the United States’ immunization strategy, emphasizing universal vaccination of infants starting at birth to curb infection rates [344]. The HBV vaccine consists of a noninfectious subunit that triggers active immunity, generating antibodies targeting the outer protein coat or surface antigen of the virus, thereby offering broad protection against all HBV genotypes (A through H) [343,345].

Extensive scientific evidence supports the notion that the hepatitis B vaccine confers long-lasting protection, with recent research affirming immunity for at least 25 years in individuals exhibiting a robust immune response from the vaccine series [346,347]. Hepatitis B, caused by HBV, can lead to both acute and chronic liver diseases, emphasizing the importance of vaccination to prevent complications such as cirrhosis and hepatocellular carcinoma [348]. HBV is a highly infectious virus transmitted through exposure to infected blood or body fluids, with perinatal and horizontal transmission being common in endemic areas [349,350]. The vaccine effectively addresses the diverse clinical phases of chronic HBV infection, contributing to a dynamic control strategy that could eventually lead to eradication [351,352]. Hepatitis B vaccination has proven instrumental in reducing the global burden of HBV infection, especially in regions with varying prevalence rates [353]. The prevalence of chronic HBV infection worldwide is estimated at 296 million people, with mortality reaching 820,000 deaths, primarily from complications such as hepatocellular carcinoma or cirrhosis [354,355].

The development of hepatitis B vaccines involved pioneering work, including the utilization of purified HBsAg obtained from carrier’ serum and subsequent advancements in recombinant technology to produce safer and more cost-effective vaccines [356,357]. The success of recombinant HBsAg in yeast-based systems has contributed to the widespread use of hepatitis B vaccines, overcoming limitations associated with plasma-derived vaccines [357,358]. While the vaccine has demonstrated excellent safety and immunogenicity, concerns about the high cost and safety of plasma-derived vaccines have led to the exploration of alternative recombinant vaccines [357]. With their diverse formulations and production methods, current hepatitis B vaccines remain crucial in the global effort to control and eventually eliminate HBV infection [359].

#### 4.4.1. Adverse Reactions

The hepatitis B vaccine has been extensively researched and established to be safe for individuals across all age groups [344]. However, it is crucial to acknowledge that the Institute of Medicine established a causal relationship between the vaccine and anaphylaxis specifically in individuals with hypersensitivity to yeast [322,344]. Zhao et al. (2020) emphasized that a documented history of anaphylaxis or serious adverse events following the initial administration of the hepatitis B vaccine is a contraindication for subsequent vaccinations. Caution is advised when vaccinating individuals with a known history of allergies to yeast [322]. Furthermore, vaccination should be postponed in individuals experiencing an acute or febrile illness [360].

Extensive studies affirm the overall safety of hepatitis B vaccines, with local reactions being the most commonly reported side effects, generally characterized as mild and transient in both children and adults. Anaphylaxis is identified as the only serious adverse event associated with hepatitis B vaccination, with an estimated incidence of 1 case at 600,000 vaccine doses [357]. Importantly, there is a lack of substantiated evidence linking the hepatitis B vaccine to other reported serious adverse events, and the incidence of serious adverse events following hepatitis B vaccination is exceedingly rare [357]. A study carried out in China, considering data from 12 years of follow-up, showed that the HBV vaccine had a safe profile, since most adverse events were mild and neurological events were relatively rare [361]. In Table 7 we summarize the rare adverse effects related to the hepatitis B vaccine [325].

#### 4.4.2. Special Populations

Hepatitis B vaccination is a critical component of public health strategies aimed at preventing and controlling HBV infection. The Advisory Committee on Immunization Practices (ACIP) recommends the vaccination of specific populations to mitigate the risks associated with contracting or experiencing complications from hepatitis B [369,370].

Vaccination is universally advised for neonates and unimmunized juveniles, with additional emphasis on cohorts identified as high-risk adults [357]. These include individuals susceptible to sexually transmitted infections, incarcerated individuals, those with hepatitis B surface antigen (HBsAg)-positive sexual partners or familial associations, men engaging in same-sex relations, intravenous drug consumers, healthcare professionals, patients undergoing dialysis, individuals aged 19 to 59 years with diabetes, those afflicted with hepatitis C, voyagers to regions endemic for hepatitis B, individuals with human immunodeficiency virus (HIV), persons suffering from chronic hepatic conditions, and individuals actively seeking safeguarding against hepatitis B [344,357]. This comprehensive approach targets diverse populations with varying risk factors, reflecting the multifaceted nature of hepatitis B transmission [371]. It is important to note that few studies have evaluated the possible relationship between the HBV vaccine and the occurrence of adverse events in individuals with HIV. However, it is believed that serious or significant adverse events are improbable [372].

Universal vaccination, starting at birth, is a cornerstone of global immunization efforts, endorsed by organizations such as the WHO [352]. This approach includes routine immunization for all infants worldwide, preventing perinatal transmission, and catch-up vaccination for individuals who do not receive the hepatitis B vaccine during infancy [373]. The catch-up vaccination is particularly prioritized for younger age groups, as they face the highest risk of chronic infection [352]. The impact of hepatitis B vaccination on reducing the disease burden is evident, with substantial decreases in HBV-related morbidity observed in vaccinated populations, especially in infants and neonates [359]. This highlights the effectiveness of early immunization in decreasing the overall incidence of the disease. Importantly, AEFI is reported less frequently in children than in adults, with the most frequent adverse events being fever and swelling [374].

## 5. New Vaccines

Recent breakthroughs in vaccine research have paved the way for the development of novel vaccines, particularly for combating global health threats. Dengue represents a significant global health challenge, with approximately 400 million infections annually, necessitating effective intervention strategies [375]. Dengue fever, an arboviral disease, primarily spreads to humans through mosquito bites, predominantly by *Aedes aegypti*, and occasionally by *Aedes albopictus* [376]. The causative agent, dengue virus (DENV), is a member of the *Flaviviridae* family and comprises four distinct serotypes: DENV-1, DENV-2, DENV-3, and DENV-4 [377]. All serotypes have the potential to infect humans, contributing to the complexity of dengue transmission and the challenges in disease control and management [378]. The most common adverse effects of the dengue vaccine include headaches, fatigue, soreness, itching, or pain at the injection site, as well as overall discomfort [379]. Recent studies have highlighted the emergence of novel dengue vaccines, including Dengvaxia^®^ (CYD-TDV) and Denvax^®^ (TAK003), with promising results in clinical trials [380,381]. Moreover, heterologous prime-boost regimens, combining inactivated vaccines with alum and live-attenuated vaccines, show potential for heightened immunogenic responses [375].

Concurrently, malaria remains a formidable threat, particularly in sub-Saharan Africa where it accounts for the majority of cases and fatalities [382]. *Plasmodium falciparum*, one of the five species responsible for human malaria, has the highest mortality rate [382]. The RTS,S/AS01 (Mosquirix^®^) vaccine has emerged as a promising tool in malaria prevention efforts, demonstrating favorable safety profiles and cost-effectiveness [383]. Recent endorsements by the World Health Organization (WHO) recommend RTS,S vaccination for children as young as 5 months in regions with moderate to high transmission rates, underscoring its potential to mitigate severe malaria burdens [384]. By inducing antibodies against the circumsporozoite protein (CSP), RTS,S vaccination offers a means to target the infective form of *Plasmodium* transmitted by mosquitoes, signifying a pivotal advancement in malaria control strategies [385,386]. After receiving a malaria vaccine, serious side effects may occur. Approximately 1% of participants may experience adverse reactions such as febrile convulsions [387]. The monkeypox (MPX) virus, an enveloped double-stranded DNA virus classified within the Orthopoxvirus genus of the Poxviridae family [388], has recently garnered increased attention due to an outbreak originating from West and Central Africa and spreading beyond endemic regions [389]. The UK reported a significant case in which a traveler who returned from Nigeria in May 2022, which promoted heightened awareness and surveillance efforts [389]. In response to the threat posed by MPX, the FDA licensed the JYNNEOS™ vaccine, a replication-deficient MVA (modified vaccinia Ankara) vaccine, for the prevention of smallpox or monkeypox disease in adults deemed at high risk for infection [390]. JYNNEOS™, also known as Imvamune^®^ or Imvanex^®^, demonstrates efficacy against orthopoxviruses, including MPX, and is a vital tool for treating outbreaks and protecting vulnerable populations [391]. Tolerable side effects, including redness, firmness/tightening, discomfort, induration, itching, sore throat, myalgia, headache, chills, and nausea, have been documented in clinical studies of the monkeypox vaccine’s injection site [392]. Despite its historical confinement to Africa, recent cases outside the continent highlight the global importance of effective vaccination strategies for mitigating the spread of MPX [393].

## 6. Perspectives and Challenges

The perspectives and challenges surrounding vaccine pharmacovigilance are multifaceted, reflecting the evolving landscape of immunization programs. The WHO defines pharmacovigilance as the science and activities related to detecting, assessing, understanding, and preventing adverse effects or any other medicine/vaccine-related problem [394]. It is recommended that population health strategies boost immunization rates, emphasizing the need to address access issues, increase community demand, and implement provider-based or system-based interventions [151]. Strict pharmacovigilance surveillance for vaccines is very important because of their unique characteristics, such as the changing frequency of preventable diseases and the potential for strain replacement [91]. The heterogeneity of stakeholders, including those opposing immunization, adds complexity to vaccine pharmacovigilance [91].

Several countries recognize the need to strengthen crucial features for effective surveillance [95]. These include risk quantification, disease burden analysis, identification of high-risk patient subgroups, and improved communication strategies, especially for high-risk groups [91]. International collaboration is deemed mandatory to enhance vaccine pharmacovigilance globally [22]. Key challenges in strengthening surveillance systems, include resource dependency, the accommodation of large numbers of reports, and early detection, investigation, and analysis of adverse events [395,396]. Challenges also include preparing for mixed-schedule vaccination and detecting local clusters of immunization error-related adverse events [22]. Effective sharing of data between vaccine manufacturers, sponsors, and regulatory authorities is essential for interpreting passive surveillance data and supplementing clinical trial safety information [130].

The initiation of pharmacovigilance during the experimentation phase of a new drug emphasizes ethical clinical trials and vigilant monitoring of adverse effects [19]. Post-marketing surveillance in Phase IV is crucial for deciphering hidden effects [397]. The Global Advisory Committee on Vaccine Safety (GACVS) has monitored new vaccines post-market authorization [398]. The GACVS characterizes and maintains the safety profile of WHO-recommended vaccines, providing advice on research priorities [93]. The WHO supports vaccines from early development through global policy recommendations, addressing challenges in production, affordability, and scalability [399].

Naniche et al. [400] stressed the need for robust pharmacovigilance systems during vaccine rollouts, including global coordination, real-time information sharing, open-source data repositories, and a strong communication component. Standardizing the reporting of AEFI and adverse events of special interest is the goal of the WHO Global Vaccine Safety Initiative [400]. These perspectives and challenges collectively underscore the ongoing efforts to ensure the safety and effectiveness of vaccines on a global scale.

## 7. Future Directions

With the incorporation of cutting-edge technology and the exploitation of extensive electronic databases, pharmacovigilance is set to undergo a substantial transition in the future [401,402]. With the use of methods like text mining and natural language processing (NLP), artificial intelligence (AI) has the potential to completely transform the evaluation of medication safety by gleaning insightful information from unstructured sources [403,404,405]. Because these techniques make it possible to identify drug–drug interactions and adverse drug reactions from textual sources, they have the potential to improve pharmacovigilance efforts [406].

A 2022 systematic review by Pilipiec et al. offered promising results for leveraging NLP in pharmacovigilance. The study found consistent evidence that NLP can effectively and accurately analyze user-generated text posted online to identify adverse drug reactions [406]. This opens doors for a new approach to pharmacovigilance, potentially improving detection and understanding of medication side effects.

In the same direction, the FDA Office of Surveillance and Epidemiology (OSE) is implementing artificial intelligence (AI) to bolster its pharmacovigilance practices. A pivotal initiative in this endeavor is the Information Visualization Platform (InfoViP), launched in 2022 [407]. This AI-powered decision support tool serves to augment the OSE’s capacity for efficient data analysis and signal detection within the ever-growing volume of adverse event reports.

Furthermore, pharmacovigilance activities such as case report input, syndromic event identification, pharmacoepidemiological investigations, data linkage, and adverse event prediction and prevention utilizing real-world data may be automated with the use of AI and machine learning [408]. Furthermore, new opportunities for tracking the safety of medications and vaccines after they are marketed as well as producing practical data to support decision-making are presented by the growth of large-scale distributed database networks [409]. Pharmacovigilance projections for 2030 made by Arlett et al. [410], include improved ICSRs collection and reporting, performance evaluation of on-market medications, and heightened patient and healthcare professional involvement. Every phase of automation seeks to increase pharmacovigilance operations’ efficiency, minimize human intervention, and streamline procedures—all of which eventually improve patient care and medication safety.

Enhanced reporting systems that streamline data collection from diverse sources, including healthcare providers, patients, and registries, will be crucial for a comprehensive understanding of vaccine safety and efficacy. Furthermore, fostering collaboration with public health agencies, research institutions, and vaccine manufacturers will facilitate the rapid identification and mitigation of potential vaccine-related risks.

## 8. Conclusions

In summary, this review explores vaccine pharmacovigilance, unraveling multifaceted challenges and promising perspectives. Strengthening surveillance systems is crucial for early detection and rapid response to adverse events. The unique characteristics of vaccines, including strain dynamics and stakeholder diversity, necessitate tailored approaches for effective risk communication. The pivotal role played by organizations underscores the importance of standardized safety protocols and research priorities. As global vaccine recommendations surge, addressing challenges in production, affordability, and scalability remains imperative.

## Figures and Tables

**Table 1:** Examples of available viral vaccines in terms of their safety and efficacy.

Virus	Platform	Overview	Reference
SARS-CoV-2	Nucleic acid, viral vector, inactivated virus, and subunit protein	Most vaccines either eliminate or probably lower the percentage of individuals who have COVID-19 symptoms that have been proven, and for some, there is strong evidence that the immunizations lessen serious or critical illness.	[54,55,56]
Poliovirus	Inactivated and live-attenuated	The efficacy of the vaccinations against the three different poliovirus types is quite good. However, there are notable variations in each vaccine’s safety and effectiveness profiles as well as how it functions. High levels of immunity are conferred by finishing the polio vaccination regimen. The usual four-dose series of inactivated polio vaccine has an effectiveness of 99% to 100% after three doses.	[57,58,59]
A(H1N1)pdm09	Inactivated and live-attenuated	When combined, all vaccines significantly decreased the prevalence of influenza in adults, children, and the elderly that was confirmed by a laboratory. Many vaccination kinds are safe for use in adults and the elderly, even if the live-attenuated vaccine proved to be more effective than the inactivated version in children.	[60,61,62]
Hepatovirus A	Recombinant	Booster doses improve the hepatitis B vaccine’s effectiveness. Strategies for both targeted and universal vaccinations substantially lower the prevalence of hepatitis B.	[63,64,65]
Varicella-zoster virus (VZV)	Recombinant and live-attenuated	Adverse effects from varicella immunization are rare and should be considered alongside its substantial benefits. Varicella vaccination has had a remarkable positive effect on public health, it has led to a reduction of over 90% in cases of hospitalizations and deaths related to varicella.	[66,67]
Vesicular stomatitis virus (VSV)	Recombinant	The vesicular stomatitis virus (VSV) is being explored as a promising candidate for antiviral vaccine development. Extensive research aims to ensure satisfactory expression of viral antigens while maintaining vector safety. Multiple VSV-based vaccines have been developed, notably including a highly effective vaccine against the Ebola virus, which recently received clinical approval.	[68,69]
Measles virus (MV)	Live-attenuated	The measles virus (MV) vaccine that is currently available on the market is not only safe but also highly effective and reasonably priced. Administered to a significant number of children, it has consistently demonstrated its efficacy and safety. Just a single dose or two low-dose of the MV vaccine are ample to provide lifelong immunity.	[70,71]

**Table 2:** Adverse reactions reporting systems of Brazil, US, Japan and European Countries.

Pharmacovigilance Tool	Country	Associated Agency	Description	References
Drug Vigilance Center (VigiMed)	Brazil	Brazilian Health Regulatory Agency (ANVISA)	Developed by The Uppsala Monitoring Centre (UMC), this is a web-based system for managing individual case safety reports (ICSR).	[109,110]
Vaccine Adverse Event Reporting System (VAERS)	US	Food and Drug Administration (FDA)	In 1990, the Vaccine Adverse Event Reporting System (VAERS) was established and implemented by the US FDA and Centers for Disease Control and Prevention (CDC) to collect reports related to adverse events associated with biological products, including vaccines.	[111,112]
EudraVigilance	European Union Countries	European Medicines Agency (EMA)	Pharmacovigilance database responsible for overseeing the collection and analysis of suspected adverse reactions to medicines authorized in the European Economic Area.	[113,114]
Medical Information for Risk Assessment Initiative (MIHARI) project	Japan	Pharmaceuticals and Medical Devices Agency (PMDA)	Objective of using large-scale electronic health information databases as innovative sources of information for pharmacoepidemiological drug safety assessments in Japan.	[115,116]
Pharmacovigilance Africa (PAVIA)	Ethiopia, Eswatini, Nigeria, and Tanzania	European Developing countries Clinical Trials Partnership (EDCTP)	Objective to enhance pharmacovigilance by leveraging collaborative support from multiple institutions both in Europe and Africa.	[117,118]
Therapeutic Goods Administration (TGA)	Australia	Australian Government Department of Health and Aged Care	Regulating medicines, medical devices, and biologicals to safeguard the health and well-being of the Australian population, ensuring that these products are both effective and safe for use.	[119,120]

**Table 3:** The COVID-19 vaccines available and the rare adverse effects related.

Developer	Platform	Indicated Ages	Rare Adverse Effects	Reference
Pfizer/BioNTech (BNT162b2/Cominarty)	mRNA	4 months to 12 years and older	Anaphylaxis; myocarditis/pericarditis	[220,223,224,225]
Moderna (mRNA-1273/Spikevax)	mRNA	6 months to 18 years and older		[226,227,228]
Novavax (NVX-CoV2373)	Recombinant protein, adjuvant	12 years and older	Possible risk of myocarditis/pericarditis	[229]
Janssen/Johnson & Johnson (Ad26.COV2.S)	Replication-incompetent adenovirus 26 vector	18 years and older	Thrombotic complications associated with thrombocytopenia; Guillain–Barre syndrome; possible risk of myocarditis/pericarditis	[230,231,232]
Pfizer/BioNTech (BA.4/BA.5 bivalent)	mRNA	12 years and older	Myocarditis/pericarditis	[233,234]
Butantan/Sinovac (Coronavac)	Inactivated virus antigen	3 years and older	Anaphylaxis; Guillain–Barre syndrome	[235,236]
Oxford/AstraZeneca (ChAdOx1 nCoV-19/Covishield)	Recombinant adenovirus vector	18 years and older	Thrombosis with thrombocytopenia syndrome (TTS); Guillain–Barre syndrome	[237,238]
Gamaleya Research Institute Sputnik V (Gam-COVID-Vac)	Adenovirus D-26 D-5	18 years and older	Immune thrombocytopenia and thrombosis	[239]

**Table 4:** Risk factors for developing severe COVID-19.

Special Group	Clinical Considerations	References
Advanced age	Advanced age is associated with an increase in the rate of hospitalizations and mortality due to COVID-19.	[248,249,250]
Obesity	Patients with body mass index (BMI) ≥40 kg/m^2^ as a high-risk condition for severe COVID-19 (obesity was associated with an increased risk of intubation or death).	[251,252]
Diabetes mellitus	The risk of mortality was higher with glycated hemoglobin (A1C) levels of 7.6 to 8.9 percent compared to 6.5 to 7 percent.	[253,254]
Hypertension	Patients with history of hypertension is associated with an increase in the rate of hospitalizations and mortality due to COVID-19.	[255,256,257]

**Table 5:** The polio vaccines available and the rare adverse effects related to them.

Platform	Strains	Rare Adverse Effects	References
bOPV	Sabin types 1 and 3	Vaccine-associated paralytic poliomyelitis	[283]
OPV and bOPV	Type 1; Types 1 and 3	Paralysis, asthma-like reaction	[284]
IPV	-	Anaphylaxis	[285]
IPV	Sabin	Thrombocytopenia, allergic purpura	[286]
IPV	Wild	Thrombocytopenia, epilepsy	

IPV: inactivated polio vaccine; OPV: oral polio vaccine; bOPV: bivalent oral polio vaccine.

**Table 6:** The influenza vaccines available and the rare adverse effects related to them.

Type	Platform	Rare Adverse Effects	References
Subtypes A and B	Inactivated	Acute disseminated encephalomyelitis (ADEM)	[326]
A(H1N1) pdm09	Inactivated	Encephalitis	[327]
-	Inactivated	Febrile seizures	[328]
A (H1N1)	Inactivated/Live-attenuated	Transverse myelitis	[329,330]
-	Inactivated/Live-attenuated	Optic neurits	[331,332]
A (H1N1)	Inactivated	Guillain–Barré Syndrome	[333,334]
Tri and quadrivalent	Inactivated	Anaphylaxis	[323,335]

**Table 7:** The hepatitis B vaccines available and the rare adverse effects related to them.

Platform	Rare Adverse Effects	References
Recombinant	Febrile convulsion	[362,363]
-	Optic neuritis	[364,365]
Recombinant	Guillain–Barré syndrome	[366,367]
Recombinant	Anaphylaxis	[368]

## Data Availability

Data are contained within the article.

## Abbreviations

ACIP	Advisory Committee on Immunization Practices
ADEM	Acute disseminated encephalomyelitis
ADR	Adverse drug reactions
AEFI	Adverse Event Following Immunization
AI	Artificial Intelligence
ANVISA	Brazilian Health Regulatory Agency
CDC	Centers for Disease Control and Prevention
CIFAVI	Interinstitutional Committee for Pharmacovigilance of Vaccines and other Immunobiologicals
CSM	Committee on the Safety of Medicines
DENV	Dengue virus
ECDC	European Centre for Disease Prevention and Control
EDCTP	European Developing countries Clinical Trials Partnership
EMA	European Medicines Agency
EU	European Union
FDA	Food and Drug Administration
GACVS	Global Advisory Committee on Vaccine Safety
GVP	Good practices in pharmacovigilance
HA	Hemagglutinin
HBV	Hepatitis B virus
HBsAg	Hepatitis B surface antigen
HIV	With human immunodeficiency virus
ICSR	Individual Case Safety Reports
ICH	International Conference on Harmonization
IIV	Inactivated influenza virus
IPV	Inactivated poliovirus vaccine
LAIV	Live-attenuated influenza vaccine
LMICs	Low- and middle-income countries
MIHARI	Medical Information for Risk Assessment Initiative
MMR	Measles, mumps, and rubella
MPX	Monkeypox
NA	Neuraminidase
NPS	National Pharmacovigilance System
OPV	Oral poliovirus vaccine
PAVIA	Pharmacovigilance Africa
PMDA	Pharmaceuticals and Medical Devices Agency
PVAE	Post-vaccination adverse events
QIV	Quadrivalent vaccines
QR	Quick response
TGA	Therapeutic Goods Administration
UMC	Uppsala Monitoring Centre
USFDA	US Food and Drug Administration
VAERS	Vaccine Adverse Event Reporting System
VDPV	Vaccine-derived poliovirus
VSV	Vesicular stomatitis virus
VZV	Varicella-zoster virus
WHO	World Health Organization
